# Letter to the editor: Measles outbreak in Gothenburg urban area, Sweden, 2017 to 2018: low viral load in breakthrough infections

**DOI:** 10.2807/1560-7917.ES.2019.24.30.1900465

**Published:** 2019-07-25

**Authors:** Shelly Bolotin, Natasha S Crowcroft, Stephanie L Hughes, Gaston De Serres

**Affiliations:** 1 Public Health Ontario, 480 University Avenue, Suite 300, Toronto, Canada; 2 Dalla Lana School of Public Health, University of Toronto, 155 College Street, Toronto, Canada; 3 Department of Laboratory Medicine and Pathobiology, University of Toronto, 1 King’s College Circle, Toronto, Canada; 4 ICES, 2075 Bayview Ave, Toronto, Canada; 5 Institut National de Santé Publique du Québec, Québec, Canada; 6 Laval University, Quebec, Canada

**Keywords:** measles, measles transmission, vaccinated cases, breakthrough cases

**To the editor:** We read with interest the study by Sundell et al. [1], describing a measles outbreak in Sweden that included breakthrough cases. The study characterised the clinical symptoms and laboratory results of each case and described differences in the clinical presentation and laboratory results for breakthrough vs naïve cases; the authors noted that no vaccinated cases transmitted measles in the outbreak. The authors concluded, based on their findings and those of other published studies, that a high risk of measles transmission is limited to unvaccinated cases and that contact tracing of vaccinated cases can likely be limited to individuals with prolonged close contact and immunocompromised individuals.

While we agree that transmission from vaccinated cases is less frequent than transmission from naïve cases, there are several reported examples of this occurring in the literature that may negate excluding them from in-depth contract tracing. Although too numerous to detail here individually, some examples include: (i) transmission in Canada in 2011 from a one-dose vaccinated case resulting in 678 cases [2], (ii) transmission in a health clinic in Israel in 2017 from a three-dose vaccinated case resulting in eight secondary cases [3], and (iii) an outbreak with several instances of measles transmission from vaccinated individuals (1–2 doses) in South Korea, in close settings e.g. in households and a school [4]. In similar circumstances, contact tracing and follow-up is important for preventing further spread and reducing morbidity.

While the authors suggest that breakthrough cases can be classified based on IgG titre and avidity alone, the number of previous doses and Ct values, which are an indication of viral load, may be more relevant measures of infectiousness [5]. In their study, the authors described widely ranging Ct values for both breakthrough and naïve cases. It is likely that for similar Ct values, vaccinated and unvaccinated cases would have similar contagiousness. Unfortunately, with so few breakthrough cases, this cannot be properly assessed. The authors also reported that breakthrough cases with one dose had more severe symptoms than cases who received two doses. For example, of 11 one-dose vaccinated cases, five (45%) were ‘confirmed cases’ according to the European Union criteria, compared with one in five (20%) for two-dose cases. Similarly, during the 2011 outbreak in Canada, only 3% (3/102) of cases who received two or more vaccine doses were hospitalised, a substantially lower proportion than unvaccinated or one-dose vaccinated cases, of whom 13% (59/465) and 21% (13/63) were hospitalised, respectively [2]. 

Breakthrough cases in one-dose recipients are often considered to be primary vaccine failures, whereas measles in two-dose recipients are more likely to be secondary vaccine failures. The persistence of some immunity in individuals with secondary vaccine failure may explain their milder clinical symptoms and may also decrease the risk of secondary transmission compared with cases with primary vaccine failure.

The number of secondary cases depends not only on the characteristics of the index case but also on the characteristics of the contacts that affect the duration and intensity of contact with the index case, vaccination status and pertinent medical conditions (such as immunosuppression). Other, population-level factors may affect the risk of onwards transmission, such as population vaccine coverage and the prevalence of immunity from a previous infection.

With so few breakthrough cases in this study, it is difficult to draw robust conclusions regarding the risk of transmission. However, transmission by vaccinated cases is well-documented. Although this risk may be smaller with two- compared with one-dose cases, more data are needed to comprehensively assess the risk and determinants of transmission from vaccinated cases before ceasing contact tracing in these scenarios.
